# A novel intron mutation in *FBN-1* gene identified in a pregnant woman with Marfan syndrome

**DOI:** 10.1186/s41065-020-00170-w

**Published:** 2021-01-06

**Authors:** Yuduo Wu, Hairui Sun, Yihua He, Hongjia Zhang

**Affiliations:** 1 Department of Cardiac Surgery, Beijing Anzhen Hospital, Capital Medical University, No.2, Anzhen Road, Chaoyang District, Beijing, 100029 China; 2 Key Laboratory of Medical Engineering for Cardiovascular Disease, Ministry of Education, Beijing, China; 3 Beijing Key Laboratory of Maternal-Fetal Medicine and Fetal Heart Disease, Beijing Anzhen Hospital, Capital Medical University, Beijing, China; 4 Ultrasound Department of Beijing Anzhen Hospital, Capital Medical University, No.2, Anzhen Road, Chaoyang District, Beijing, 100029 China

**Keywords:** Marfan syndrome, *FBN-1* gene, Splicing mutation, Mini gene, Sequencing

## Abstract

Marfan syndrome (MFS) is one of the most common hereditary connective tissue diseases, with great individual heterogeneity. We reported a Chinese pregnancy with Clinical diagnosis of MFS, performed whole-exome sequencing, and screened for the genetic abnormality. We also conducted an in vitro mini-gene splicing assay to demonstrate the predicted harmful effects of an intronic variant of *FBN-1*. Exome sequencing identified a novel intronic variant (c.6497-13 T>A) in intron 53 of the *FBN-1* gene (NM_000138.4). It’s predicted to insert 11 bp of intron 53 into the mature mRNA. The mini-gene splicing experiment demonstrated that c.6497-13 T>A could result in 11 bp retention in intron 53 to exon 54 (c.6496_6497ins gtttcttgcag) and the use of an alternative donor causing the frameshift p.Asp2166Glyfs*23. According to the results, the pregnant woman chose to continue the pregnancy and gave birth to a healthy baby. This study expands the genetic mutation spectrum of MFS patients and indicates the importance of intron sequencing.

**Supplementary Information:**

The online version contains supplementary material available at 10.1186/s41065-020-00170-w.

## Introduction

Marfan syndrome (MFS) is an autosomal dominant connective tissue disease with an incidence of 2–3/10000 without any ethnic, geographic, or professional orientation [1]. The condition caused by mutations in the human fibrillin-1 (*FBN-1*) gene [2]. More than 3000 *FBN-1* site mutations have been reported. The mutation spectrum is composed of nonsense, missense, frameshift, and exon deletion, etc. [3] Among them, about 90% of MFS patients’ mutation sequence information limited to the coding region and specific splice sites. However, about 10% of patients still cannot identify the genetic cause of the disease [1]. Part of the reason may be that advances in sequencing have not significantly affected the clinical interpretation of uncertain significance variants, such as intron variants that may affect splicing [4, 5]. This study, reports a pregnant woman with a clinical diagnosis of MFS, who carried a novel intron mutation (NM_000138.4:c.6497-13 T>A). Through the in vitro characterization of the effect of this mutation on mRNA, we determined that it was a pathogenic mutation and used it for the patient’s family management, and successfully gave birth to a normal baby. The report is as follows.

## Case presentation

### Patient information

**Fig. 1:**
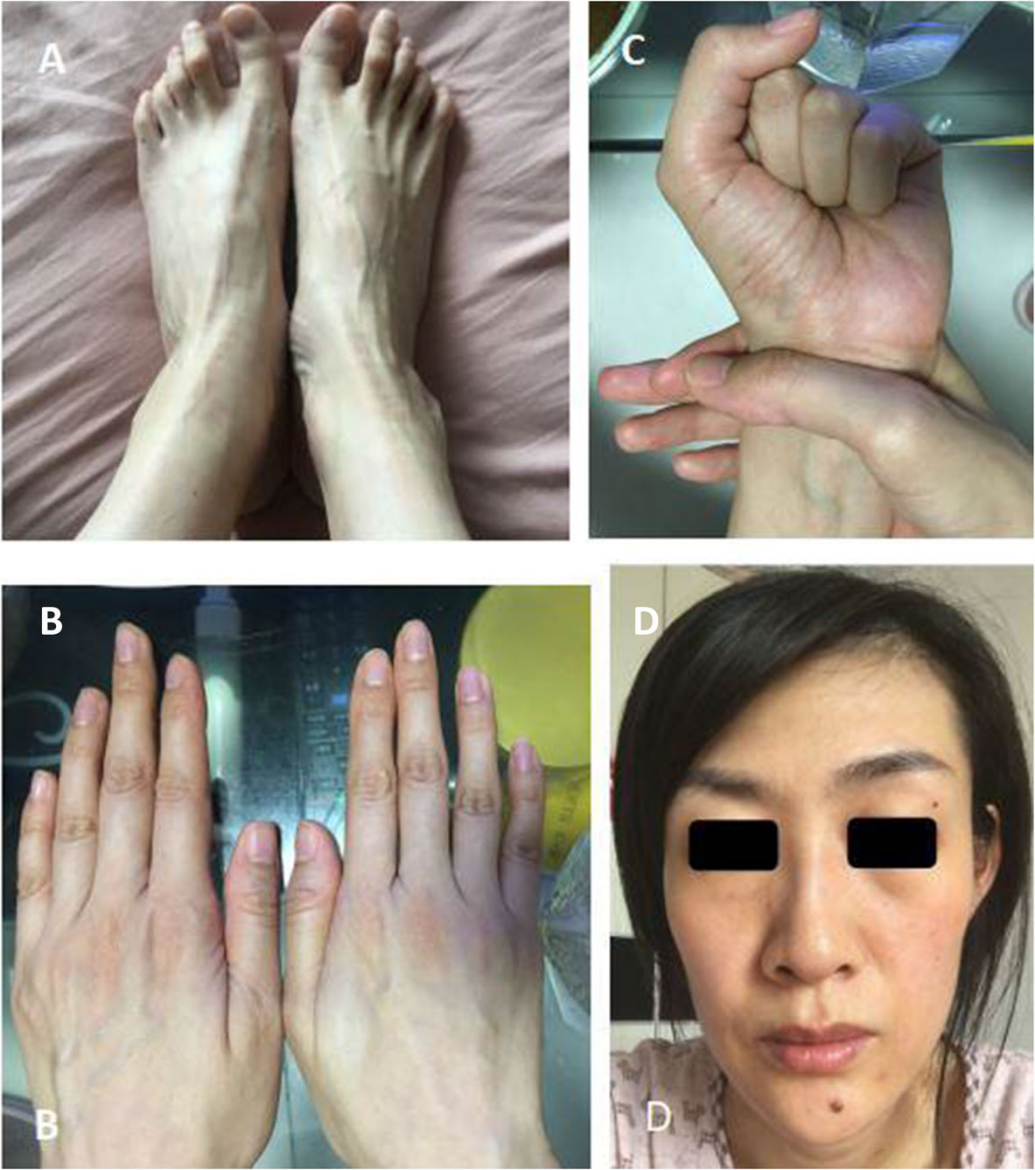
Arachnodactyly of the proband (**a** and **b**); positive wrist (**c**); characteristic face with dolichocephaly, malar hypoplasia, enophthalmos, myopia more than 3 diopters (**d**)

**Fig. 2:**
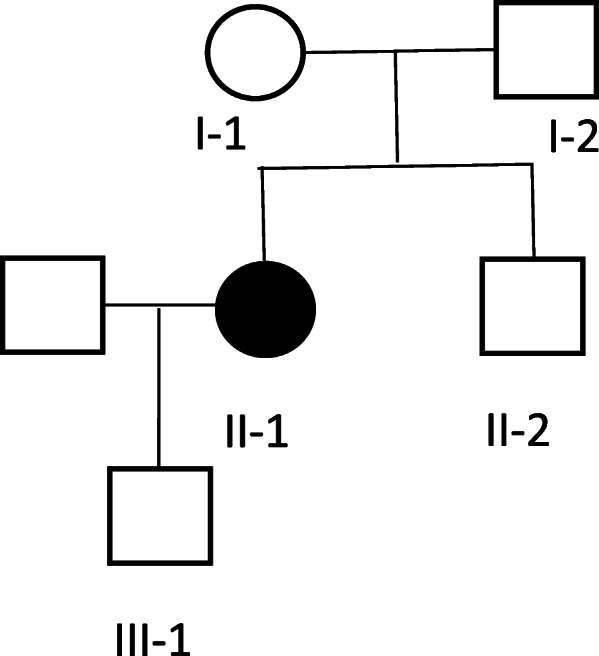
Family diagram of the proband, I-1, I-2: parents of the proband; II-1: proband; II-2: younger brother of the proband; III-1: son of the proband

Proband female: 33 years old, height 183 cm, weight 60 kg, the patient admitted to the cardiac surgery department of our hospital because of a sudden aortic dissection, and the physical examination found: slender limbs, positive wrist signs, thoracic deformity, high myopia, as shown in Fig. 1. Ultrasound examination revealed: widening of the aortic root, mitral valve prolapse, clinical suspected MFS, surgical treatment, no genetic testing. Last year, the patient was pregnant and had another outpatient consultation. Family status: The family has three generations, five people, and one patient (the proband herself), as shown in Fig. 2. The proband’s parents and younger brother have no clinical symptoms of MFS, and both parents have no relatives with relevant symptoms.

### Molecular findings

**Fig. 3:**
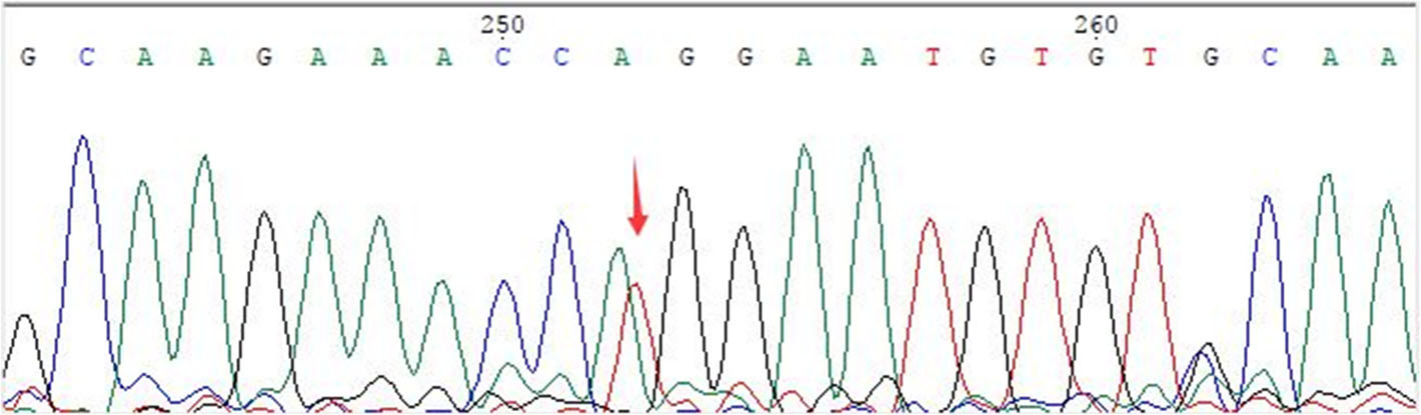
Sanger sequence diagram of the proband, the red arrow points to the mutation site. Polymerase chain reaction (PCR) amplication was performed using forward primer (caactcctgtgagctgttgc) and reverse primer (acgttgtccacagtgagtcc). The obtained sequence was compared with the *FBN-1* reference gene (NM_000138.4) to identify mutations

A trio (proband and her parents) whole-exome sequencing was performed using methods described previously [6]. Whole-Exome sequencing identified a novel variant, c.6497-13 T>A, in intron 53 of the *FBN-1* gene (NM_000138.4). Subsequent Sanger sequencing confirmed that the mutation was heterozygous in the pregnant woman (Fig. 3) and wild-type in her parents, younger brother, and fetus.

**Fig. 4:**
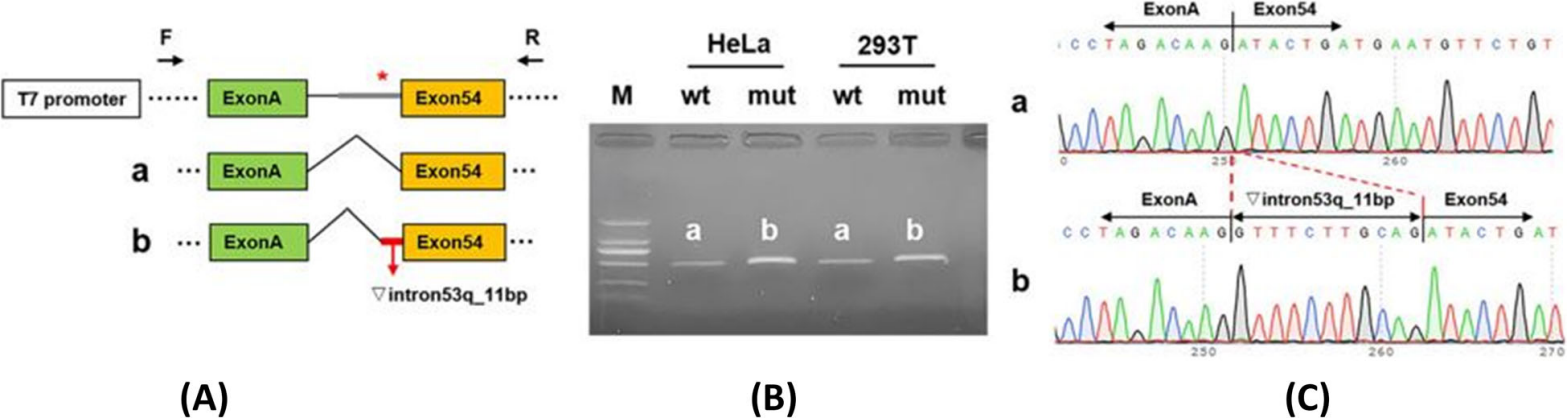
Results of the mini-gene splicing assay. **a** Schematic diagram of mini-gene construction and the c.6497-13 T>A variant related abnormal splicing. The asterisk indicates the location of the c.6497-13 T>A variant. **b** Gel electrophoresis of RT-PCR products: the band of the mutant was bigger than the wild-type. **c** Mini-gene product sequencing results: **a** The wild-type mini-gene (FBN1-wt) formed normal mRNA composed of exon54; **b** The mutant mini-gene (FBN1-mut) caused a splicing abnormality, resulting in the retention of the 11 bp in intron 53

### In silico splicing analysis

Computational predictions conducted using HSF (http://www.umd.be/HSF3/HSF.html) revealed that the intronic c.6497-13 T>A variant might influence the splicing process by differentially affecting canonical versus cryptic splice site utilization. The newly detected variant predicted to insert 11 bp of intron 53 into the mature mRNA (Fig. 4-a). This variant should give rise to a frameshift and a premature termination codon (p.Asp2166Glyfs*23).

### Results of the mini-gene splicing assay

To prove that the c.6497-13 T>A variant caused the 11 bp insertion, we then conducted the mini-gene splicing experiment using RNA from blood lymphocytes as described previously [7]. The mini-gene splicing products were analyzed by PCR amplification with plasmid-specific primers and visualized with polyacrylamide gel electrophoresis. The electrophoresis results of wild-type and c.6497-13 T>A transfections indicated that the mutant band was bigger than the wild-type, and the migration became slower (Fig. 4-b). The amplicons confirmed by Sanger sequencing showed that the cDNA fragment obtained from the c.6497-13 T>A plasmid contained an additional 11 bp in intron 53 to exon 54 (c.6496_6497ins gtttcttgcag) (Fig. 4-c). This result is consistent with the in silico analysis result.

### Screening of other genes associated with thoracic aortic aneurysm and dissection

Besides finding the *FBN-1* mutation presented above, we also analyzed variants in genes associated with thoracic aortic aneurysm and dissection (Supplementary Table 1). No pathogenic/likely pathogenic variants were identified in these genes.

## Discussion

This study describes a pregnancy with Clinically diagnosed MFS that carried a novel intronic variant in the *FBN-1* gene. We added this novel variant to the *FBN-1* mutational repertoire and demonstrated this variant’s effect at the mRNA level by in vitro Mini-gene splicing assay. Furthermore, Based on the results of genetic tests, pregnancy decisions are guided.

This novel intronic variant in the *FBN-1* gene (c.6497-13 T>A) has previously not been reported as pathogenic or benign and has not observed in the general population (gnomAD: https://gnomad.broadinstitute.org). It showed a deleterious effect by multiple in silico algorithms. Furthermore, in vitro experiment of mini-gene splicing assay verified that the variant alters splicing, leading to a frameshift and a premature termination codon. It is a loss-of-function variant and expected to result in either an abnormal truncated protein product or loss of protein from this allele through nonsense-mediated mRNA decay. Following the revised Ghent nosology for the MFS, the c.6497-13 T>A variant was pathogenic. In this work, we highlighted the importance of including extended intronic regions in analysizing WES (whole-exome sequencing, WES) results during variant prioritization and post-analytical phase for clinical purposes. This work also points out the need to introduce functional investigations to verify further the pathogenicity of intronic variants with a potential pathogenic effect.

Through this study, we also found that the diagnosis of MFS through genetic testing has limitations. Despite advances in genetic testing technology, about 10% of patients still cannot be diagnosed through genetics. According to our case and some previous scattered case reports, it’s suggeste that part of the reason is the variation of introns or other non-coding regions [8]. On the other hand, it is necessary to consider the differentiation from other hereditary macrovascular diseases, such as: Loeys-Dietz Syndrom, Ehlers-Danlos Syndrom, etc. [9] We found no relevant mutations in the case.

For MFS patients with atypical clinical phenotypes, genetic testing can not only diagnose the disease but also distinguish it from other genetic macrovascular diseases with similar phenotypes. Through large-scale testing within the family, it is also possible to find patients with gene mutations without clinical phenotypes and help them prevent adverse aortic events in advance. At the same time, it can also provide fertility guidance for women of childbearing age. Like the pregnant women in this study, she performed amniocentesis in the second trimester. The genetic test results showed negative, and the pregnancy continued, giving birth to a healthy baby.

Because both parents are not the carrier for the mutation, the proband’s mutation should be a de novo mutation in the family. Although the proband gave birth to a healthy baby, if this patient becomes pregnant again in the future, the probability of MFS in the second child is still 50% [1]. Therefore, we recommended that this part of patients undergo amniotic fluid puncture during the second trimester of pregnancy and check the karyotype.

In conclusion, this study enlarges the mutation spectrum of *FBN-1* and points out the importance of intronic sequence analysis and the need for integrated functional studies in *FBN-1* diagnosis. The literature on *FBN-1* intronic variants affecting non-typical splice sites is currently limited to single reports [10]. Therefore, the large sample size and large cohort study need to carry out. To further reveal the significance of non-coding region (intron) variation to MFS.

## Supplementary Information

**Additional file 1: Supplementary Table 1.** Genes associated with thoracic aortic aneurysm and dissection.

## Data Availability

Availably.

## Abbreviations

MFS: Marfan syndrome
WES: whole exome sequencing
PCR: Polymerase chain reaction
